# Parents' Attitudes toward Oral Rehydration Therapy in Children with Mild-to-Moderate Dehydration

**DOI:** 10.1155/2013/828157

**Published:** 2013-11-04

**Authors:** Vered Nir, Erez Nadir, Yaffa Schechter, Adi Kline-Kremer

**Affiliations:** ^1^Department of Pediatrics, Hillel Yaffe Medical Center, 38100 Hadera, Israel; ^2^Department of Neonatology, Hillel Yaffe Medical Center, 38100 Hadera, Israel

## Abstract

*Objective*. According to current guidelines, the first line of treatment for mild-to-moderate dehydration is oral rehydration; the second line is rehydration through a nasogastric tube. Both methods are widely underused. This study was conducted to evaluate parents' attitudes towards rehydration methods used in pediatric emergency departments. *Design*. 100 questionnaires were distributed to parents of children who visited emergency room due to gastroenteritis and suspected dehydration. *Results*. 75 of the parents expected their child to get IV fluids. 49 of them would refuse to consider oral rehydration. 75 of the parents would refuse to consider insertion of nasogastric tube. Parents whose children were previously treated intravenously tended to be less likely to agree to oral treatment. Parents were more prone to decline oral rehydration if the main measurement of dehydration was the child's clinical appearance, clinical appearance with vomiting, or child's refusal to drink and were more likely to agree if the main measurement was diarrhea, diarrhea with clinical appearance, or clinical personnel opinion. * Conclusions*. This is the first study to examine parents' expectations. We found that in the majority of cases, parents' expectations contradict current guidelines. Efforts should be taken to educate parents in order to allow full implementation of the guidelines.

## 1. Introduction

Acute gastroenteritis is a common condition among children, especially under 5 years of age. It is a major cause of morbidity, comprising up to 16% of emergency department visits [1]. The major complication is dehydration. The treatment of choice in mild-to-moderate dehydration is oral rehydration therapy (ORT). A program by the world health organization that promoted the use of oral rehydration solution at outpatient level decreased mortality rate from diarrhea by 75% from 1980 to 2008 worldwide [2]. Oral rehydration is as effective as intravenous fluid rehydration in cases of mild-to-moderate dehydration [3]. The risk of treatment failure is about 4% [4], yet it eliminates the risk of intravenous treatment complications such as infection at the sight of insertion [5]. Certain studies even found lower risk of major complications such as seizures and even mortality [4]. The most prevalent cause of treatment failure is persistence of vomiting [6].

The second line of treatment for dehydration is rehydration through a nasogastric tube. That method was found to be as good as intravenous rehydration even in moderate-to-severe dehydration [7]. It was proven that nasogastric tube rehydration does not last longer time and carries less complications [8]. It was also found that blood studies taken during the insertion of venous access do not indicate the hydration status more efficiently than clinical examination, so drawing blood is not justified for cases of clinical mild dehydration [8]. 

It is well known that oral rehydration is being underused considering the recommendations. Up to 75% of the centers treating children who have gastroenteritis worldwide use intravenous rehydration as the first line of treatment [4]. Parents whose child was previously treated for dehydration tend to expect the same treatment the next time [1]. A study that examined the treatment preferences among pediatric emergency care fellowship directors found that they underused oral rehydration because they thought oral rehydration requires more time. They also felt that both parents and the referring physicians expected intravenous treatment [9].

Guidelines that contrast the common practice are difficult to implement. The literature for obtaining such a change is sparse. An implementation program that was conducted in Wales and included a number of teaching sessions to medical and nursing staff found improvement in using oral and nasogastric tube rehydration, and the change was maintained over 10 years [10].

No previous study known to the authors systematically evaluated parents' attitudes towards rehydration methods in pediatric emergency departments.

In this study, we found that in the majority of cases parents expected IV fluids rehydration, which contradict current guidelines.

## 2. Methods

Questionnaires were distributed to parents of children who reported to the Pediatric Emergency Department in Hillel Yaffe Medical Center in Hadera from August until November 2012 due to gastroenteritis and suspected mild-to-moderate dehydration. Parents whose children showed severe dehydration or parents whose children were diagnosed with chronic gastrointestinal disease such as Crohn's disease were excluded.

The parents were asked what made them think their child was dehydrated, what kind of treatment they had expected, what treatment the child received, whether they had reported to an emergency room previously for suspected dehydration, and what treatment they had received on the previous occasion. The parents were also asked as for their willingness to consider oral dehydration therapy and insertion of a nasogastric tube for rehydration.

As all data was nonparametric, we used the *χ* ^2^ test to compare it between groups. Statistical significance was set at a *P* value ≤0.05. All calculations were made using IBM SPSS software version 20 (International Business Machines Corp., New Orchard Road, Armonk, NY, USA). The study was approved by local board for human research. Verbal informed consent was obtained from all parents before fulfilling the questionnaire. 

## 3. Results

One hundred questionnaires were collected. Ninety-seven of the children were completely healthy; otherwise, 1 child was previously diagnosed to have autism, 1 child had Down's syndrome, and 1 child was in the process of medical investigation for suspected epilepsy. The characteristics of the patients are summarized in Table 1.

Among parents of children who were previously treated intravenously, there was a tendency not to agree to oral treatment. There was no difference in their willingness to consider a nasogastric tube. 

*χ* ^2^ analysis was performed between every available pair of questions in the questionnaire. There was a statistically significant connection only between parents' expectations and actual treatment, consideration of PO fluids and dehydration characteristics, and consideration of PO fluids and actual treatment.

There was a tendency for parents' willingness to consider oral rehydration according to the reason that made them think their child was dehydrated. Parents were more prone to decline oral rehydration if the main measurement of dehydration was clinical appearance, clinical appearance in combination with vomiting or child's refusal to drink. Parents were more prone to agree to oral rehydration if the main reason that caused them to think their child was dehydrated was diarrhea, diarrhea in combination with clinical appearance or clinical personnel opinion. 

When parents expected IV rehydration, usually actual treatment was IV rehydration.

Also, oral rehydration was the actual treatment only if parents were willing to consider it.

## 4. Discussion

Acute gastroenteritis accompanied by mild-to-moderate dehydration is a common cause of emergency department visits around the world. Oral rehydration is the first line of treatment according to current guidelines. The second line of treatment should be the giving of rehydration solution through a nasogastric tube. These methods are being underused according to current reports [4]. It was found that clinical personnel felt that parents expected intravenous rehydration therapy [9]. This is the first study to examine parents' expectations. 

Our findings show that in the majority of cases parents' expectations contradict current guidelines. 50% of the parents refused to consider oral rehydration and 75% of the parents refused to consider insertion of a nasogastric tube. The current study shows that in our area parents' expectations upon reporting to the emergency department due to suspected dehydration may burden the implementation of the guidelines. 

We also found that similar to previous studies [1] parents to children who were previously treated intravenously were less likely to consider oral rehydration.

Oral rehydration is being underused in our pediatric emergency department, as in most medical centers worldwide. This is therefore a trend that enforces itself. Efforts should be made by medical staff to educate parents about the recommended treatment for dehydration in order to allow implementation of the guidelines and thus provide better medical treatment for their children.

A major limitation to our study is its small size. A larger study must be conducted in order to support our findings. Further studies should be conducted in order to assess the best measures to educate parents about the current clinical guidelines.

## Figures and Tables

**Table 1:** Characteristics of the patients.

Sex	Male	58
	Female	42

Age (years)	0-1	18
	1–3	33
	3–9	22
	9–17	27

Dehydration characteristic	Vomiting	41
	Clinical	25
	Vomiting + clinical	10
	Referring physician	7
	Diarrhea	6
	Lack of drinking	3
	Diarrhea + clinical	2
	Outpatient nurse	1
	Do not know	5

Parents' expectation	IV fluids	75
	Investigation	1
	Do not know	24

Actual treatment	IV fluids	85
	PO rehydration	15

Past ER treatment	IV fluids	24
	PO fluids	3
	Do not remember	2
	No past ER refer	71

May have considered PO fluids	Yes	50
	No	49
	Do not know	1

May have considered gavage	Yes	18
	No	75
	Do not know	7
